# Single-phase Fe_0.5_Co_0.5_S_2_ cathode material for long working time thermal batteries

**DOI:** 10.1039/d6ra06872c

**Published:** 2026-09-14

**Authors:** Hui Yang, Hai Zhou, Xinlu Li, Shaoming Qiao, Qibing Wu, Min Kang

**Affiliations:** a College of Materials Science and Engineering, Chongqing University Chongqing 400044 China lixinlu@cqu.edu.cn; b State Key Laboratory of Advanced Chemical Power Sources Zunyi 563006 China km20056570@163.com; c Department of Chemistry and Chemical Engineering, Zunyi Normal University Zunyi 563006 China

## Abstract

Multi-metal disulfides are widely employed as cathode materials for thermal batteries owing to their high electronic conductivity, high specific capacity, and exceptional stability of the cathode–separator interface. However, conventional synthesis methods for multi-metal disulfides suffer from low production efficiency. Herein, we report a scalable route to synthesize Fe–Co binary disulfide (Fe_0.5_Co_0.5_S_2_) *via* high-temperature solid-state sintering of a mixture of Fe–Co alloy and sublimed sulfur. The resulting Fe_0.5_Co_0.5_S_2_ possesses single-phase, high thermal stability, and low resistance, which effectively enhance its discharge capacity in thermal batteries. At a current density of 100 mA cm^−2^ and a discharge temperature of 460 °C, Fe_0.5_Co_0.5_S_2_ delivers a high specific capacity of 595.4 mAh g^−1^ with a cutoff voltage of 1.5 V. Notably, the specific capacity decreases by only 10.7% at a discharge temperature of 540 °C, indicating an extended working temperature range. As a result, the voltage upon constant and pulse current loading of Fe_0.5_Co_0.5_S_2_ in a 102 V thermal battery is obviously higher than that of the mechanically mixed FeS_2_/CoS_2_. This work provides a simple and efficient strategy for the preparation of binary disulfides for thermal batteries.

## Abstract 2

Single-phase Fe_0.5_Co_0.5_S_2_ with excellent thermal stability and conductivity is prepared *via* high temperature sulfurization of Fe–Co alloy. Fe_0.5_Co_0.5_S_2_ delivers a stable operating voltage and excellent pulse performance in a 102 V thermal battery.

## Graphical abstract

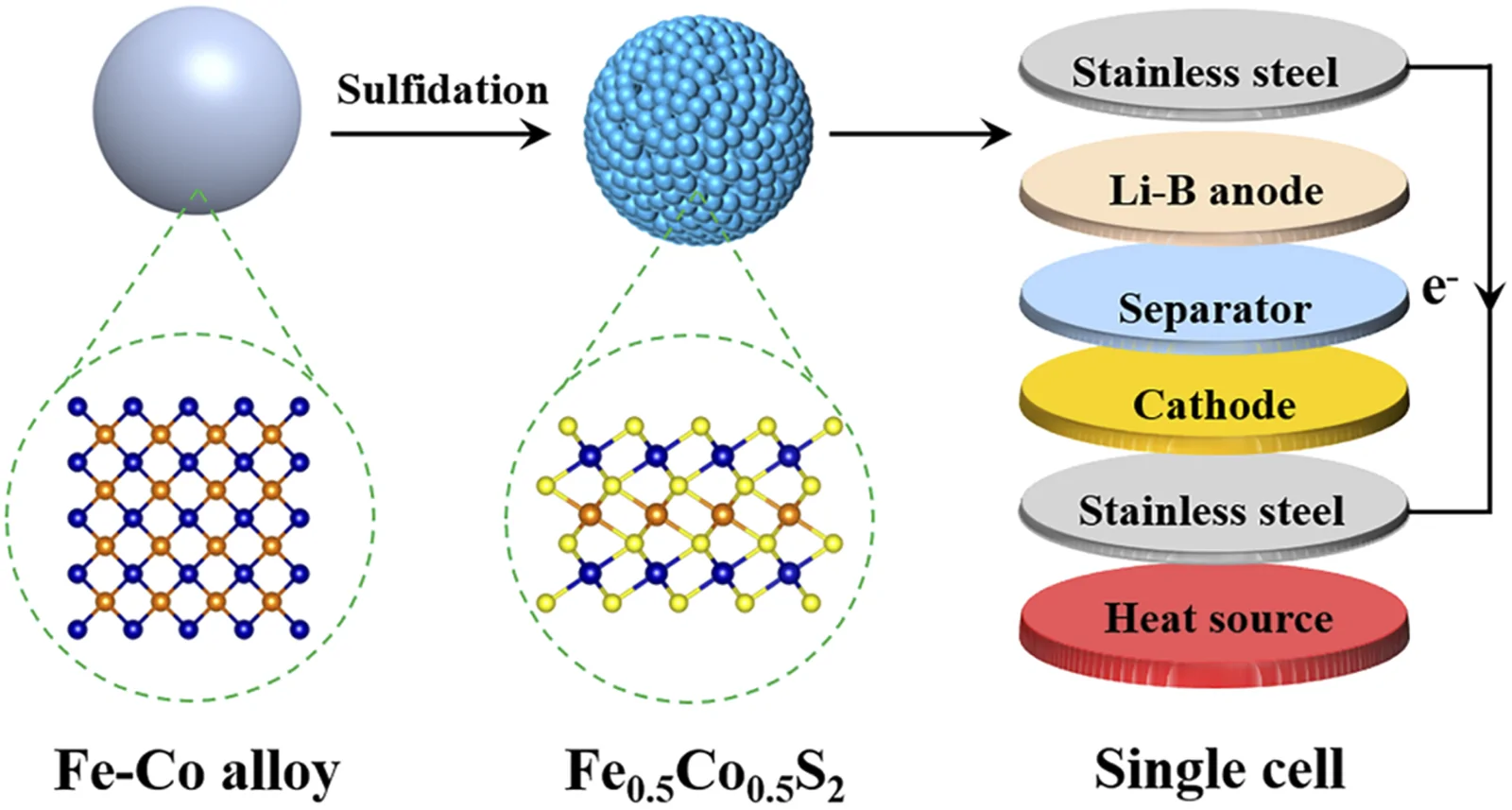

## 1 Introduction

The thermal battery is one kind of primary power source, which is widely used in aerospace equipment, aircraft ejection seats, *etc.* Thermal batteries can be activated rapidly (generally in less than 1 s) after the ignition of their pyrotechnic system by an external signal, during which the non-conductive molten salt is heated and melted into an ionic conductor.^1–3^ Thermal batteries have the advantages of high power and energy density, compact structure, long storage life, being maintenance-free and so on.

Transition metal disulfides, such as FeS_2_, CoS_2_, and NiS_2_, are the main cathode materials for thermal batteries, owing to their superiorities of exceptional thermal stability, outstanding electronic conductivity, high specific capacity, and excellent stability of the cathode-separator interface.^1–4^ Different disulfides have their own advantages in terms of electronic conductivity, thermal stability, and voltage platform. For example, compared with CoS_2_, FeS_2_ has a slightly higher voltage platform, poorer thermal stability, and lower electronic conductivity.^1–3^ As a consequence, composites of disulfides are generally used as cathode materials for thermal batteries, in order to take advantage of each mono-disulfide.

Hydrothermal method can effectively control the morphology and particle size of materials.^5,6^ It is generally used for the synthesis of heteroatom-doped surface-modified disulfides, such as Co_*x*_Ni_1−*x*_S_2_,^7–9^ MoS_2_-CoS_2_,^10^ Co_4_S_3_ nanotube,^11^ and MoS_2_.^12^ Meanwhile, carbon modified disulfides can also be prepared *via* hydrothermal synthesis to improve the electronic conductivity.^13,14^ These materials generally possess excellent thermal battery performances, such as low polarization and high specific capacity. For instance, Co-doped NiS_2_ delivered a specific capacity of 434.8 mAh g^−1^ at 100 mA cm^−2^ with a cutoff voltage of 1.5 V, and a high energy density of 142.5 Wh kg^−1^ was achieved in a 66 ± 10 V thermal battery at 100 mA cm^−2^.^7^ However, the production efficiency of hydrothermal synthesis is very low. High temperature thermal sintering of a mixture of transition metal and sublimed sulfur can also be used to prepare mono-metal^15^ and multi-metal disulfides (*e.g.* heteroatom-doped NiS_2_ (ref. 16 and 17) and Ni_0.5_Co_0.5_S_2_).^18^ However, long-time ball-milling is required for the synthesis of disulfide composites to ensure the homogeneous dispersion of different metals.

Meanwhile, disulfide composites can be directly synthesized *via* high temperature thermal sintering of a mixture of metal alloy and sublimed sulfur.^19,20^ In our previous work, FeNiCoS_2_ ternary disulfide was prepared *via* the high temperature sulfurization of Fe–Ni–Co alloy.^19^ The resulting FeNiCoS_2_ showed a high specific capacity of 601 mA h g^−1^ at a current density of 200 mA cm^−2^ and a discharge temperature of 460 °C with a cutoff voltage of 1.5 V. However, an obvious capacity loss (21.1%) was observed when the cell was discharged at 540 °C, due to the poor thermal stability of this ternary disulfide. This cathode material cannot meet the needs of long working time thermal batteries. As a thermally activated primary battery, the working time of a thermal battery largely depends on its thermal life, because it can supply energy only when the electrolyte is molten.^21–23^ The internal temperature of long working time thermal batteries is higher than that of conventional batteries, so as to ensure a longer thermal life. However, high temperature can lead to the decomposition of cathode materials with poor thermal stability,^13,24^ the generated sulfur vapor will react with the Li–B anode and produce a large amount of heat, which can lead to battery runaway.

In this work, Fe_0.5_Co_0.5_S_2_ binary disulfide with a single phase is prepared *via* the direct sulfurization of Fe–Co alloy. The present Fe_0.5_Co_0.5_S_2_ possesses the superiorities of high thermal stability, low resistance, and high specific capacity in thermal batteries. Especially, the capacity decrease is only 10.7% when the discharge temperature rises from 460 to 540 °C, demonstrating excellent performance at high working temperatures. Furthermore, the performance of Fe_0.5_Co_0.5_S_2_ is investigated in a 102 V thermal battery, which demonstrates excellent long-time working ability and pulse loading capability. This work provides a simple and efficient strategy for large-scale preparation of Fe–Co binary disulfides with higher thermal battery performances than FeS_2_, CoS_2_, and the mechanically mixed FeS_2_/CoS_2_.^19,25^

## 2 Experimental

### 2.1 Synthesis of materials

Fe–Co alloy with a metallic molar ratio of 1 : 1 was used as the starting material to synthesize Fe_0.5_Co_0.5_S_2_. Fe–Co alloy and sublimed sulfur with a weight ratio of 1 : 3 were mixed using a mortar and pestle. The mixture was heated to 400 °C under Ar with a heating rate of 5 °C min^−1^ and maintained at 400 °C for 4 h. The resultant sample was further heated at 500 °C for 2 h under Ar to remove excess sulfur.

FeS_2_ and CoS_2_ were also prepared *via* a similar method using Fe and Co powder as the metal source, respectively. The FeS_2_/CoS_2_ composite was obtained *via* mechanical mixing of FeS_2_ and CoS_2_ powders with a molar ratio of 1 : 1.

### 2.2 Thermal battery assembling and testing

The 0.25 g cathode and 0.45 g separator were pressed into a cathode/separator composite pellet with a diameter of 20 mm under 20 MPa. A Li–B pellet (60 wt% Li) with a thickness of 0.5 mm was used as the anode. Single cells were tested on an IT8511 plus single-channel programmable electronic load. Detailed information for the material characterization, single-cell fabrication, and test methods is presented in the SI.

Thermal batteries with a peak voltage of 102 V were also manufactured by using Fe_0.5_Co_0.5_S_2_ and FeS_2_/CoS_2_ as cathode materials, respectively. A total of 3.6 g cathode material and 3.3 g separator were pressed into a cathode/separator composite pellet with a diameter of 66 mm. A Li–B pellet (60 wt% Li) with a thickness of 0.35 mm was used as the anode. A pressed Fe/KClO_4_ pellet was used as the internal pyrotechnic source. By using the typical thermal battery structure, the single cells were sealed in a stainless steel housing with a diameter of 78 mm and a height of 180 mm, forming a 102 V full thermal battery. The battery was activated and discharged at ambient temperature.

## 3 Results and discussion

### 3.1 Material characterization

**Fig. 1:**
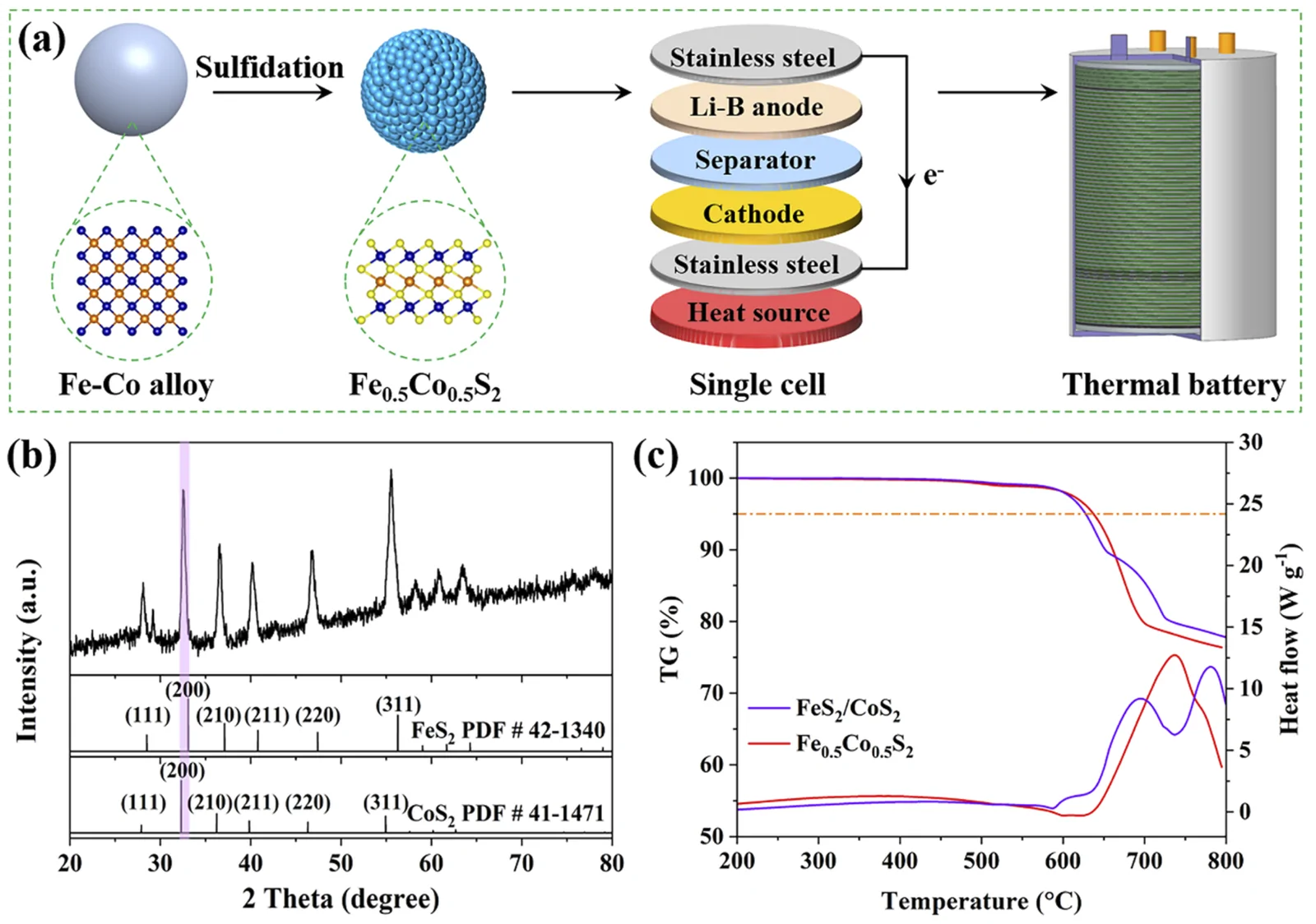
(a) Schematic illustration of the synthesis process of Fe_0.5_Co_0.5_S_2_, and the structure of a single cell and a full thermal battery. (b) XRD pattern of Fe_0.5_Co_0.5_S_2_, (c) TG and DSC curves of Fe_0.5_Co_0.5_S_2_ and FeS_2_/CoS_2_ under Ar from room temperature to 800 °C with a heating rate of 10 °C min^−1^.

Fig. 1(a) presents the synthesis process of Fe–Co binary disulfide, and the structure diagram of a single cell and a full thermal battery. The material is prepared *via* high temperature sulfurization of Fe–Co alloy with a metallic molar ratio of 1 : 1, which is then used as cathode material for thermal batteries. As shown in Fig. S1, well-crystallized FeS_2_ and CoS_2_ are obtained *via* the high temperature sulfurization process using Fe and Co powder as the metal source, respectively. Accordingly, Fe–Co alloy can also be vulcanized by sulfur producing binary disulfide, and a complete mutual substitution of Fe and Co can be realized in a wide atomic concentration range due to the isomorphic feature of FeS_2_ and CoS_2_.^26^ X-ray diffraction (XRD) pattern of Fe–Co alloy is shown in Fig. S2, the diffraction peaks centered at 44.8 and 65.3° can be ascribed to the (110) and (200) crystal planes of Fe–Co alloy (PDF #44-1433). Fig. 1(b) shows the XRD pattern of the obtained material. The diffraction peaks are located between the characteristic diffraction peaks of FeS_2_ (PDF # 42-1340) and CoS_2_ (PDF # 41-1471), indicating a single-phase structure. Considering that Fe and Co will not be lost during the solid-state reaction and can be completely vulcanized by excessive sulfur, the composition of the resulting material should be Fe_0.5_Co_0.5_S_2_.

The phase structure of the prepared samples was also analyzed by XRD Rietveld refinement, using a face-centered cubic crystal structure model. The results are shown in Fig. S3 and S4. The fitted pattern of Fe_0.5_Co_0.5_S_2_ possesses a low weighted profile factor (*R*_wp_, 2.63%), suggesting a high credible fitting result (Fig. S3). The fitted unit cell parameter of Fe_0.5_Co_0.5_S_2_ is *a* = *b* = *c* = 5.4660 Å, which is between the fitted parameters of FeS_2_ (5.4163 Å) and CoS_2_ (5.5321 Å), confirming the formation of single-phase Fe_0.5_Co_0.5_S_2_. The ionic radius of Fe^2+^ (0.061 nm) is close to that of Co^2+^ (0.065 nm),^27^ which is beneficial for the mutual substitution of Fe and Co to generate single-phase Fe_0.5_Co_0.5_S_2_. The uniform substitution can efficiently modify the electronic structure of Fe and Co atoms in Fe_0.5_Co_0.5_S_2_,^16,19,27^ enhancing the electronic conductivity of Fe_0.5_Co_0.5_S_2_. As a result, Fe_0.5_Co_0.5_S_2_ is expected to possess excellent thermal battery performances, such as high electronic conductivity and high specific capacity.

The thermal stability of cathode material is crucial for thermal batteries. The decomposition of disulfides decreases the capacity of cathode materials; the generated sulfur vapor reacts with the Li–B anode and produces a large amount of heat, which can make the heat of the battery run out of control. The thermal stability of Fe_0.5_Co_0.5_S_2_ and FeS_2_/CoS_2_ was tested by thermogravimetric (TG) analysis, as shown in Fig. 1(c). The TG curves of the two samples almost coincide with each other when the temperature is below 600 °C. Even so, the 5% mass loss temperature of Fe_0.5_Co_0.5_S_2_ (637.4 °C) is 10 °C higher than that of FeS_2_/CoS_2_, which is also higher than those of many other reported disulfides.^16,27,28^ Meanwhile, the differential scanning calorimetry (DSC) curve of FeS_2_/CoS_2_ displays two obvious endothermic peaks, which indicates that the main mass loss of FeS_2_/CoS_2_ can be divided into two stages, corresponding to the thermal decomposition of FeS_2_ and CoS_2_. For comparison, Fe_0.5_Co_0.5_S_2_ possesses only one endothermic peak located between the two peaks of FeS_2_/CoS_2_, which indicates that Fe_0.5_Co_0.5_S_2_ has one decomposition process, further confirming the single-phase structure. The decomposition temperature of CoS_2_ is considerably higher than that of FeS_2_,^1,2^ implying that Co–S bond possesses higher strength than Fe–S bond. As a result, the replacement of Fe–S bonds in FeS_2_ by Co–S bonds can improve the thermal stability of the Fe_0.5_Co_0.5_S_2_ solid solution.

**Fig. 2:**
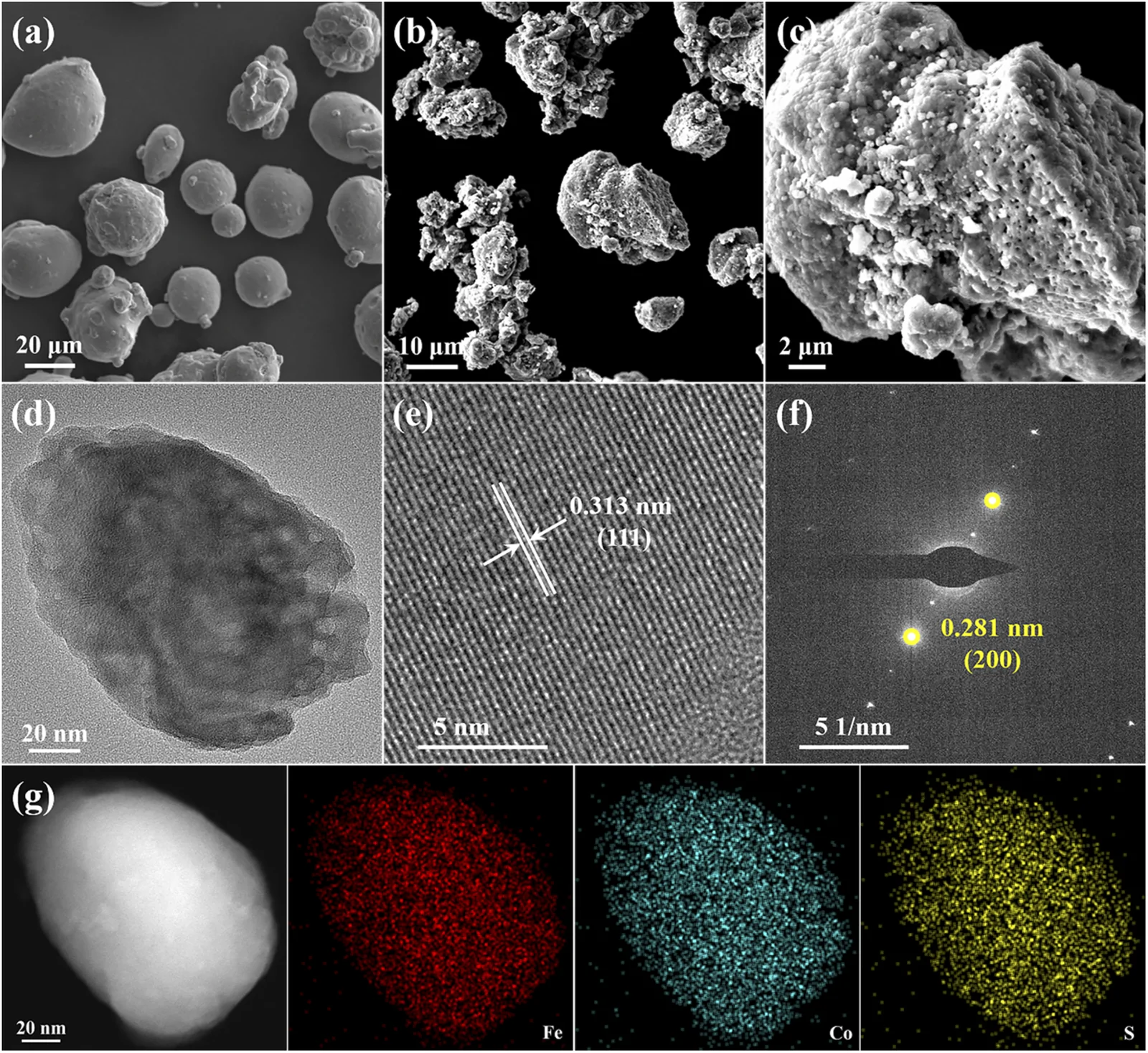
SEM images of (a) Fe–Co alloy and (b and c) Fe_0.5_Co_0.5_S_2_ prepared *via* a high-temperature thermal sintering process. (d) TEM image, (e) HRTEM image, (f) SAED pattern, and (g) TEM EDS mapping images of Fe_0.5_Co_0.5_S_2_.

The scanning electron microscope (SEM) image of Fe–Co alloy is presented in Fig. 2(a), which indicates that the alloy is composed of microspheres with diameter of 10–30 µm. The SEM energy dispersive spectroscopy (EDS) mapping images confirm the uniform distribution of Fe and Co in the alloy (Fig. S5), the atomic ratio of Fe to Co is 1.06 : 1. After the high temperature sulfurization process, the resulting Fe_0.5_Co_0.5_S_2_ consists of irregular particles aggregated by small particles, forming a porous structure with a rough surface (Fig. 2(b and c)). Similar morphology is observed for FeS_2_ and CoS_2_ (Fig. S6).^17,19^ Small particles can shorten the transmission path of electrons and ions, and the porous structure can provide abundant diffusion channels for electrolyte, both of which help to improve the electrochemical performance.^29^ SEM EDS mapping images reveal the homogenous distribution of Fe, Co, and S elements throughout the Fe_0.5_Co_0.5_S_2_ particles (Fig. S7).

The crystal structure of Fe_0.5_Co_0.5_S_2_ was also investigated by transmission electron microscopy (TEM). As shown in Fig. 2(d), the microparticle of Fe_0.5_Co_0.5_S_2_ displays an irregular sphere with a diameter of less than 150 nm. The clear and well-arranged lattice fringes in the high-resolution TEM (HRTEM) image confirm the good crystallinity of Fe_0.5_Co_0.5_S_2_ (Fig. 2(e)). The lattice fringe spacing of 0.313 nm can be ascribed to the (111) plane of Fe_0.5_Co_0.5_S_2_. The selected area electron diffraction (SAED) pattern further verifies the single-crystal feature of Fe_0.5_Co_0.5_S_2_ (Fig. 2(f)). Moreover, TEM EDS mapping images suggest the uniform distribution of different elements at the atomic scale in Fe_0.5_Co_0.5_S_2_ (Fig. 2(g)). The above results suggest that Fe_0.5_Co_0.5_S_2_ with excellent thermal stability and a single-phase structure can be obtained *via* the sulfurization of Fe–Co alloy.

**Fig. 3:**
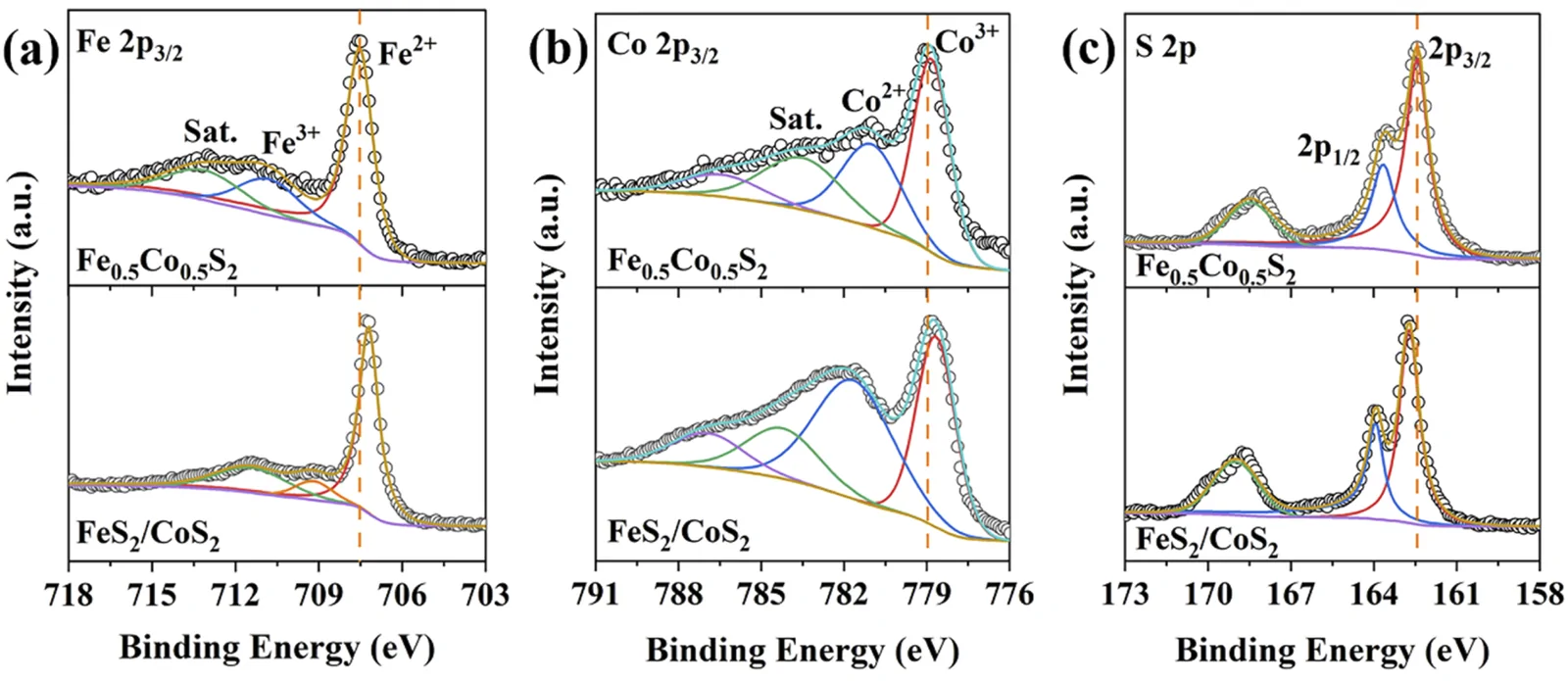
(a) Fe 2p_3/2_, (b) Co 2p_3/2_, and (c) S 2p spectra of Fe_0.5_Co_0.5_S_2_ and mechanically mixed FeS_2_/CoS_2_.

The chemical state and bonding environment of Fe_0.5_Co_0.5_S_2_ and FeS_2_/CoS_2_ were analyzed by X-ray photoelectron spectroscopy (XPS). Fe 2p_3/2_ spectra of Fe_0.5_Co_0.5_S_2_ and FeS_2_/CoS_2_ are fitted into three peaks, which can be assigned to Fe^2+^, Fe^3+^, and the satellite peak (Fig. 3(a)).^30,31^ Obviously, the Fe^2+^ and Fe^3+^ peaks of Fe_0.5_Co_0.5_S_2_ shift to higher binding energies as compared to those of FeS_2_/CoS_2_. Co 2p_3/2_ spectra of the two samples can be deconvoluted into four peaks corresponding to Co^3+^, Co^2+^, and the satellite peaks (Fig. 3(b)).^8,15,32^ The Co^3+^ characteristic peak of Fe_0.5_Co_0.5_S_2_ also shows a positive shift of 0.4 eV. S 2p spectra of the two samples can be fitted into three peaks centered at 162, 164, and 167 eV, corresponding to S_n_^2−^ (2p_3/2_), S_n_^2−^ (2p_1/2_), and S–O, respectively (Fig. 3(c)).^16,17^ Notably, the S_n_^2−^ peak of Fe_0.5_Co_0.5_S_2_ shows a negative shift of 0.3 eV compared to that of FeS_2_/CoS_2_. It should be noted that FeS_2_/CoS_2_ is the mechanical mixture of FeS_2_ and CoS_2_, thus, the chemical properties of Fe and Co in FeS_2_/CoS_2_ cannot be changed obviously in comparison to the corresponding mono-disulfides. The above analysis suggests that the outer-shell electrons of Fe and Co in Fe_0.5_Co_0.5_S_2_ are greatly regulated by mutual substitution forming an electron-defect state, which is more capable of accepting electrons than the mono-disulfides and/or mechanically mixed samples. As a result, Fe_0.5_Co_0.5_S_2_ is expected to deliver excellent thermal performances, such as low resistance and high specific capacity.^33–35^

### 3.2 Single cell performance

**Fig. 4:**
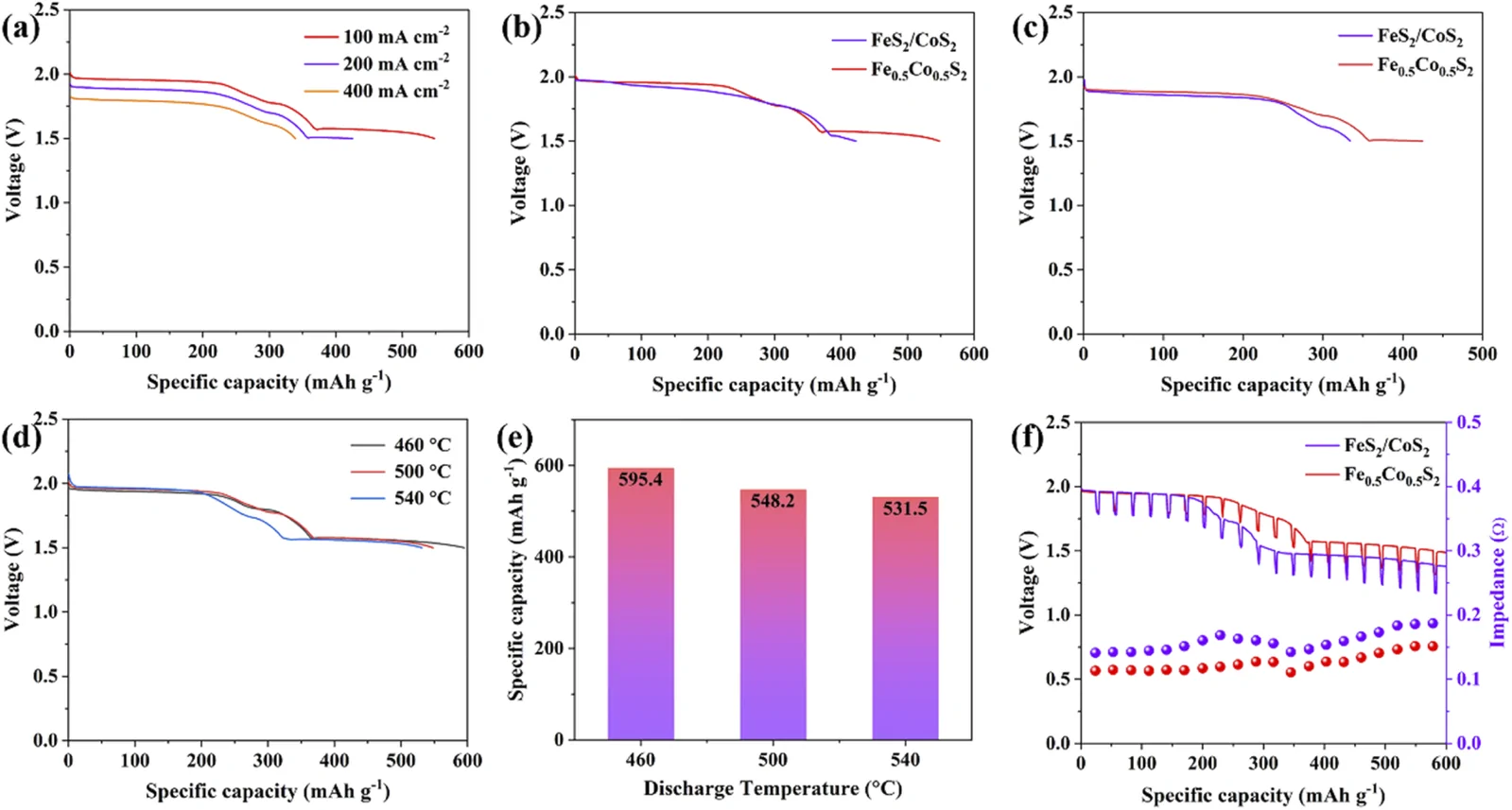
(a) Discharge curves of Fe_0.5_Co_0.5_S_2_ at different current densities. Discharge curves of Fe_0.5_Co_0.5_S_2_ and FeS_2_/CoS_2_ at (b) 100 mA cm^−2^ and (c) 200 mA cm^−2^. (d) Discharge curves and (e) specific capacity of Fe_0.5_Co_0.5_S_2_ at different temperatures. The capacity was calculated by the mass of electroactive material with a cut-off voltage of 1.5 V. (f) Pulse discharge curves and resistance of Fe_0.5_Co_0.5_S_2_ and FeS_2_/CoS_2_ at a constant current density of 100 mA cm^−2^ and a pulse current density of 500 mA cm^−2^ for 2 s every 30 s.

The thermal battery performance of Fe_0.5_Co_0.5_S_2_ was firstly evaluated by single cells, using Fe_0.5_Co_0.5_S_2_ and Li–B as the cathode and anode materials, respectively. Fig. 4(a) shows the discharge curves of Fe_0.5_Co_0.5_S_2_ at different current densities and a discharge temperature of 500 °C. Similar to many other disulfides,^19,27^ Fe_0.5_Co_0.5_S_2_ displays two obvious voltage plateaus. The voltage of the single cell decreases with increasing current density, due to the internal resistance of the cell. The calculated specific capacity of Fe_0.5_Co_0.5_S_2_ reaches 548, 425, and 339 mAh g^−1^ at current densities of 100, 200 and 400 mA cm^−2^, respectively, with a cutoff voltage of 1.5 V. Fig. 4(b) and (c) compare the discharge curves of Fe_0.5_Co_0.5_S_2_ and FeS_2_/CoS_2_ at current densities of 100 and 200 mA cm^−2^. It is noteworthy that the discharge curve of Fe_0.5_Co_0.5_S_2_ is significantly prolonged compared to that of FeS_2_/CoS_2_, and the specific capacity of Fe_0.5_Co_0.5_S_2_ at 200 mA cm^−2^ is 1.27 times that of FeS_2_/CoS_2_. It also outperforms the corresponding mono-disulfides (FeS_2_ and CoS_2_)^19^ and many other reported disulfides (Table S1). As a result, Fe_0.5_Co_0.5_S_2_ displays a greatly improved utilization of cathode material at high current densities, which is conducive to maintaining high energy density at a high power density.

To elucidate the discharge mechanism of Fe_0.5_Co_0.5_S_2_, XRD patterns of the material after discharge were collected at different cutoff voltages, as presented in Fig. S9. Upon discharge to 1.8 V, the characteristic diffraction peaks of Fe_0.5_Co_0.5_S_2_ vanish, while new diffraction peaks corresponding to Li_2_FeS_2_, Co_9_S_8_, and FeCo alloy emerge. When the cutoff voltage is further lowered to 0 V, the diffraction peaks of Li_2_FeS_2_ and Co_9_S_8_ disappear, accompanied by a notable increase in the intensity of the FeCo alloy peaks. These observations indicate that Li_2_FeS_2_ and Co_9_S_8_ serve as intermediate discharge products and are eventually converted into FeCo alloy. Based on these findings, the discharge mechanism of Fe_0.5_Co_0.5_S_2_ can be proposed as follows:^1,2^Fe_0.5_Co_0.5_S_2_ + Li → Li_3_Fe_2_S_4_ + Co_3_S_4_ + Li_2_SLi_3_Fe_2_S_4_ + Co_3_S_4_ + Li → Li_2_FeS_2_ + Co_9_S_8_ + Li_2_SLi_2_FeS_2_ + Co_9_S_8_ + Li → Fe + Co + Li_2_SFe + Co → CoFe

The influence of discharge temperature on the thermal battery performance of Fe_0.5_Co_0.5_S_2_ was also investigated in the temperature range of 460–540 °C at 100 mA cm^−2^. As shown in Fig. 4(d), similar discharge curves are obtained at different temperatures. At a relatively low temperature (460 °C), the specific capacity of Fe_0.5_Co_0.5_S_2_ reaches 595.4 mAh g^−1^ with a cutoff voltage of 1.5 V (Fig. 4(e)). Due to thermal decomposition of the cathode material, the first voltage plateau shortens slightly with increasing discharge temperature, resulting in a decreased specific capacity. Even so, Fe_0.5_Co_0.5_S_2_ shows a high specific capacity of 531.5 mAh g^−1^ at 540 °C; the capacity loss is only 10.7% compared to the value obtained at 460 °C. For comparison, the capacity of FeS_2_/CoS_2_ is 476.1 mAh g^−1^ and 359.8 mAh g^−1^, respectively, at 460 °C and 540 °C, corresponding to a capacity retention of 75.4% (Fig. S8). The significant capacity loss of FeS_2_/CoS_2_ can mainly be ascribed to the decomposition of FeS_2_. The above results confirm that Fe_0.5_Co_0.5_S_2_ can still work normally at a temperature where ordinary materials are prone to failure, demonstrating an extended operating temperature range.

The internal resistance of Fe_0.5_Co_0.5_S_2_ and FeS_2_/CoS_2_ was further investigated by pulse discharge tests. The cell was discharged at 100 mA cm^−2^ for 28 s, after which a pulse with a current density of 500 mA cm^−2^ and a width of 2 s was applied. The pulse was repeatedly applied for 20 times. The internal resistance (*R*, Ω) was calculated based on the equation *R* = (*V*_1_ − *V*_2_)/(*I*_2_ − *I*_1_),^18,19^ where *V*_1_ (V) and *I*_1_ (A) were the background voltage and current, *V*_2_ (V) and *I*_2_ (A) were the pulse voltage and current, respectively. The discharge curves and resistance are presented in Fig. 4(f). Notably, Fe_0.5_Co_0.5_S_2_ possesses a small and stable resistance with no obvious fluctuation or increase; the average value is 0.125 Ω, which is 0.023 Ω lower than that of FeS_2_/CoS_2._ Meanwhile, the internal resistance of Fe_0.5_Co_0.5_S_2_ is obviously lower than that of FeS_2_, even close to that of CoS_2_.^19^ The low resistance can enhance the transfer rate of electrons and promote the utilization of electroactive material, resulting in excellent thermal battery performance. The improved conductivity of Fe_0.5_Co_0.5_S_2_ can be ascribed to the following two reasons: (1) the mutual substitution between Fe and Co atoms in Fe_0.5_Co_0.5_S_2_ can optimize the conduction band and generate more charge carriers.^36^ (2) More importantly, the replacement of Fe atoms with Co facilitates the conversion of FeS_2_ from semiconductor to semimetal, significantly improving the electronic conductivity.^26,37^

### 3.3 Thermal battery performance

**Fig. 5:**
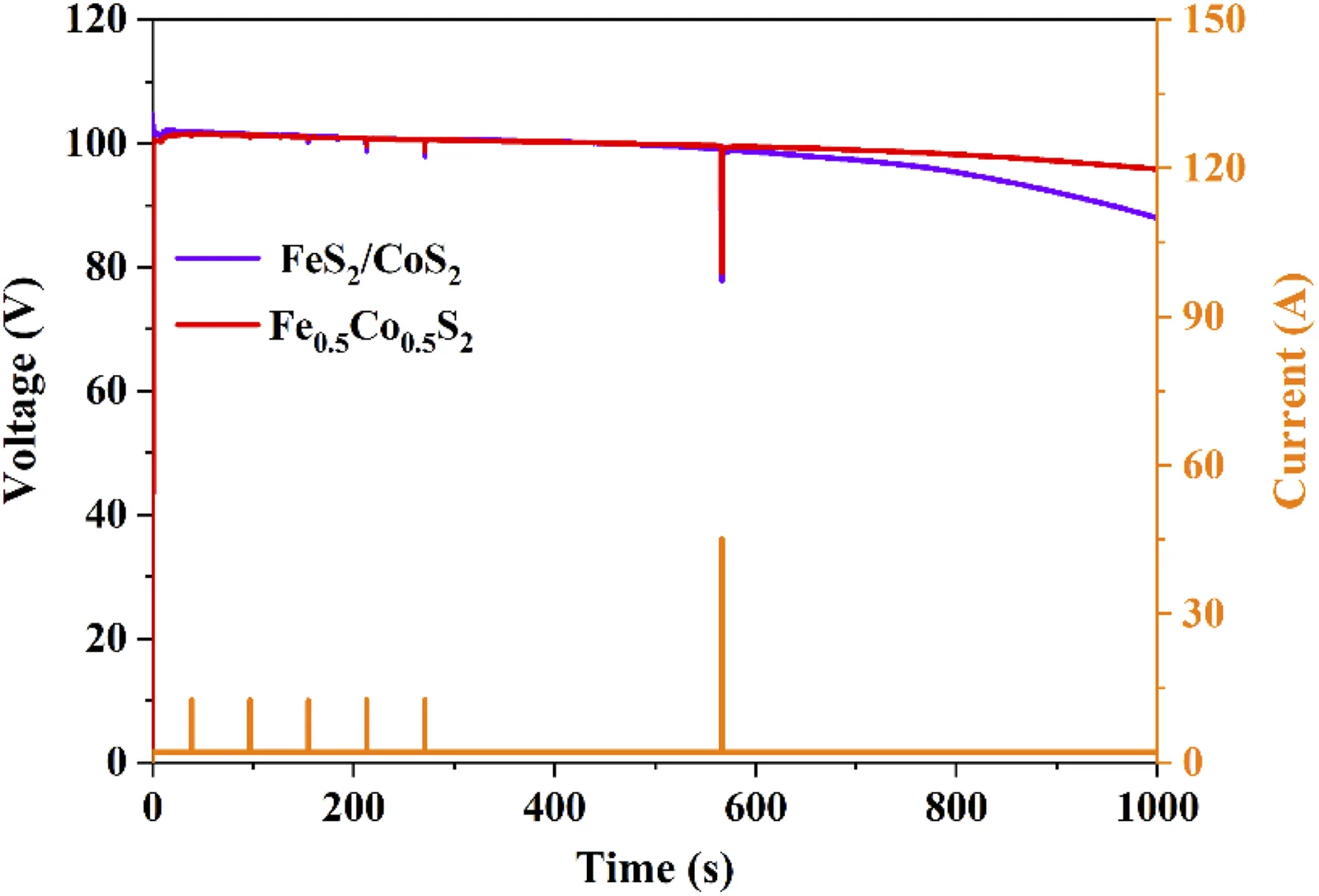
Discharge curves of the 102 V full thermal batteries at a constant current of 2.0 A (58 mA cm^−2^), and a series of pulse currents of 12.6 A (368 mA cm^−2^) and 45 A (1316 mA cm^−2^).

The single cell performance suggests that single-phase Fe_0.5_Co_0.5_S_2_ has the advantages of high thermal stability, low resistance, high specific capacity and wide operating temperature range. Thus, full thermal battery with an operating voltage of 102 V was assembled to investigate the capability of Fe_0.5_Co_0.5_S_2_ in long working time battery. For comparison, FeS_2_/CoS_2_ was also used as cathode material to fabricate the battery. The battery was discharged at a constant current of 2.0 A for 1000 s, and a series of pulse currents with values of 12.6 A and 45 A was applied. The result is shown in Fig. 5. The peak voltage of the two batteries is consistent, and the discharge curves almost coincide with each other before 500 s. Owing to its high electronic conductivity, the battery using Fe_0.5_Co_0.5_S_2_ as the cathode material displays smaller voltage drop upon the application of pulse current. Impressively, the voltage of the battery using Fe_0.5_Co_0.5_S_2_ as the cathode material is very stable during the entire discharge process, decreasing by only 5.6%. By contrast, the voltage of the other battery starts to decrease after 500 s, and an obvious voltage drop is observed in the later operating time, resulting in a low voltage retention (86.2%).

Thermal batteries are one kind of high-temperature battery, which can work normally only when the molten electrolyte is melted into an ionic conductor. As a consequence, the internal temperature of long working time thermal batteries is higher than that of conventional batteries to ensure a longer thermal life. FeS_2_/CoS_2_ is the mechanical mixture of FeS_2_ and CoS_2_, FeS_2_ with a relatively low decomposition temperature can decompose gradually during the long time high temperature discharge process, leading to capacity loss, voltage drop, and an increase in internal resistance. This is unacceptable for thermal batteries with the requirement of high voltage accuracy, especially for the battery that needs to apply large pulse in the later working stage. More importantly, the generated sulfur vapor will react with the Li–B anode in the sealed metal housing and produce a large amount of heat, which might cause battery runaway in the worst case.

By contrast, Fe_0.5_Co_0.5_S_2_ can deliver high capacity retention at 540 °C (Fig. 4(e)), implying that the decomposition of Fe_0.5_Co_0.5_S_2_ can be neglected in the full thermal battery. As a result, the voltage of the battery can remain stable for a long time. More importantly, the internal temperature of the battery will not increase significantly due to limited side reactions, and thus the battery safety can be guaranteed, especially during the unload process after activation. In addition, the high electronic conductivity of Fe_0.5_Co_0.5_S_2_ also helps to stabilize the battery voltage and improve the pulse current loading capacity. As a result, the performance of Fe_0.5_Co_0.5_S_2_ is significantly higher than that of conventional FeS_2_/CoS_2_ in long working time thermal batteries.

## 4 Conclusions

Single-phase Fe_0.5_Co_0.5_S_2_ is successfully prepared *via* the high temperature solid-state reaction between Fe–Co alloy and sublimed sulfur. Owing to its improved thermal stability and electronic conductivity compared to mechanically mixed FeS_2_/CoS_2_, the present Fe_0.5_Co_0.5_S_2_ exhibits excellent thermal battery performances, such as low resistance and high specific capacity. Notably, Fe_0.5_Co_0.5_S_2_ delivers a high specific capacity of 595.4 mAh g^−1^ at a current density of 100 mA cm^−2^ and a discharge temperature 460 °C with a cutoff voltage of 1.5 V. Meanwhile, a high capacity retention of 89.3% is also obtained at the discharge temperature of 540 °C, demonstrating excellent performance over a wide operating temperature range. As a result, the voltage of the full thermal battery using Fe_0.5_Co_0.5_S_2_ as the cathode material is very stable during the discharge process, showing a voltage decrease of only 5.6% after 1000 s. In this work, a simple and efficient strategy is developed for the large-scale preparation of high performance Fe–Co binary disulfides, which is of great significance for practical application in long working time thermal batteries.

## Conflicts of interest

The authors declare that they have no known competing financial interests or personal relationships that could have appeared to influence the work reported in this paper.

## Supplementary Material

RA-OLF-D6RA06872C-s001

## Data Availability

The authors confirm that the data supporting the findings of this study are available within the article and its supplementary information (SI). Supplementary information: XRD of FeS_2_, CoS_2_, and Fe-Co alloy; XRD Rietveld refinement of Fe_0.5_Co_0.5_S_2_, FeS_2_, and CoS_2_; SEM EDS-mapping of Fe-Co alloy and Fe_0.5_Co_0.5_S_2_; SEM of FeS_2_ and CoS_2_; single cell performance of FeS_2_/CoS_2_; XRD patterns of Fe_0.5_Co_0.5_S_2_ after discharge; thermal battery performances of various materials. See DOI: https://doi.org/10.1039/d6ra06872c.

## References

[cit1] Masset P. J., Guidotti R. A. (2008). J. Power Sources.

[cit2] Masset P. J., Guidotti R. A. (2008). J. Power Sources.

[cit3] Li C., Liu H., Wu J., Li C., Shao X., Xie G., Luo Y. (2024). J. Power Sources.

[cit4] Meng X., Liu H., Bi S., Fan S., Cao L., Yi T., Li X. (2024). J. Energy Storage.

[cit5] Bhardwaj R. K., Jayanthi S., Adarakatti P. S., Sood A. K., Bhattacharyya A. J. (2020). ACS Appl. Mater. Interfaces.

[cit6] Cong J., Luo S., Liu X., Wang Y. (2026). J. Alloys Compd..

[cit7] Guo H., Tang L., Tian Q., Chu Y., Shi B., Yin X., Huo H., Han X., Yang C., Wang C., Tang K., Wang C., Zhang X., Wang J., Kong L., Lu Z. (2020). ACS Appl. Mater. Interfaces.

[cit8] Meng X., Liu H., Bi S., Yang C., Fan S., Cao L. (2023). Electrochim. Acta.

[cit9] He Y., Cao L., Yuan G., Fan S., Li Q., Bi S., Luo C., Liu H. (2020). Ionics.

[cit10] Guo Y., Chen H., Wang C., Zheng X., Zhu Y., Jiao S. (2023). J. Power Sources.

[cit11] Wei X., Li K., Zhu D., Pinna N., Zhu Y., Quan T. (2024). J. Energy Storage.

[cit12] Zheng X., Zhu Y., Sun Y., Jiao Q. (2018). J. Power Sources.

[cit13] Xie S., Deng Y., Mei J., Yang Z., Lau W.-M., Liu H. (2017). Electrochim. Acta.

[cit14] Xie S., Deng Y., Mei J., Yang Z., Lau W.-M., Liu H. (2016). Composites, Part B.

[cit15] Hu J., Wang X., Guo H., Yang M., Yuan J., Chu Y., Zhao L. (2022). Energy Technol..

[cit16] Zhang C., Fu L., Yao B., Zhu J., Yang W., Li D., Zhou L. (2023). ACS Appl. Mater. Interfaces.

[cit17] Zhang C., Yang W., Yao B., Tang L., Tang J., Zhu J., Yang W., Zhou L., Pan Z., Fu L. (2024). J. Energy Storage.

[cit18] Chen X., Zhang C., Yao B., Tang L., Yuan Z., Zhu J., Yang W., Zhou L., Fu L. (2024). ACS Appl. Mater. Interfaces.

[cit19] Kang M., Qiao S., Yang H., Zhao Z., Tang L., Fan Z., He Z., Zhou H. (2025). Energy Technol..

[cit20] Xu Y., Li H., Cao Y., Wang C., Cui Y. (2025). RSC Adv..

[cit21] Guidotti R. A., Masset P. (2006). J. Power Sources.

[cit22] Masset P., Guidotti R. A. (2007). J. Power Sources.

[cit23] Bu X., Wei X., Quan T., Zhu Y. (2024). Energy Fuels.

[cit24] Yang W., Zhou L., Luo Z., Zhu J., Yang W., Li D., Fu L. (2020). Adv. Eng. Mater..

[cit25] Geng J., Zhu Y., Wu Q. (2020). Acta Armamentarii.

[cit26] Clamagirand J. M., Ares J. R., Flores E., Diaz-Chao P., Leardini F., Ferrer I. J., Sánchez C. (2016). Thin Solid Films.

[cit27] Tang L., Zhang C., Guo H., Zhao H., Tian Q., Wang J., Pan Z., Meng J., Tang J., Zhou L., Chen C., Fu L. (2023). Electrochem. Commun..

[cit28] Xu C., Jin C., Gong X., Wang X., Xie S., Fan Y. (2022). J. Energy Storage.

[cit29] Adarakatti P. S., Kempahanumakkagari S. K. (2018). Electrochemistry.

[cit30] Tian Q., Yu Z., Wu Y., Liu H. (2022). J. Mater. Sci.: Mater. Electron..

[cit31] Huang R., Luo S., Sun Q., Qian L., Yan S. (2026). ACS Energy Lett..

[cit32] Yang P., Zhang X., Cao Y., Xie Y., Wang C., Li X., Cui Y. (2024). Appl. Energy.

[cit33] Zhou H., Wen P., Chen X., Zhao N., Wang Q., Ma Z., Kang M. (2024). J. Power Sources.

[cit34] Adarakatti P. S. , in Fundamentals of Electrochemistry, 2025, 1496, 17–46

[cit35] Tian G., Sun Y., Li Z., Wei F., Zeng C., Sun J., Zhang C. (2026). AIChE J..

[cit36] Hakami J. (2026). Int. Commun. Heat Mass.

[cit37] Kinner T., Bhandari K. P., Bastola E., Monahan B. M., Haugen N. O., Roland P. J., Bigioni T. P., Ellingson R. J. (2016). J. Phys. Chem. C.

