# Supplementary material for: Single-phase Fe0.5Co0.5S2 cathode material for long working time thermal batteries
Source: RSC Adv. 2026 Sep 14;16(44):54769–75. doi: 10.1039/d6ra06872c (PMC13572918; doi:10.1039/d6ra06872c)
Supplement: Supplementary file 1 [file RA-016-D6RA06872C-s001.pdf]

Supplementary Information (SI) for RSC Advances.
This journal is © The Royal Society of Chemistry 2026

Supporting Information

Single phase $Fe_{0.5}Co_{0.5}S_2$ cathode material for long working time thermal batteries

Hui Yang,[1,2] Hai Zhou,[3] Xinlu Li,[1*] Shaoming Qiao,[2] Qibing Wu,[2*] Min Kang[2*]

[1] College of Materials Science and Engineering, Chongqing University, Chongqing, 400044 China

[2] State Key Laboratory of Advanced Chemical Power Sources, Zunyi, 563006, China

[3] Department of Chemistry and Chemical Engineering, Zunyi Normal University, Zunyi, 563006, China

Material characterization

The morphology of samples was investigated by scanning electron microscope (SEM, Quanta200, FEI) and transmission electron microscope (TEM, JEM 2100F, JEOL). Energy dispersive spectroscopy (EDS) mapping and selected area electron diffraction (SAED) pattern were also carried out in conjunction with TEM. The crystal structure of materials was characterized by X-ray diffraction (XRD) on a X'pert (Malvern Panalytical) diffractometer. X-ray photoelectron spectroscopy (XPS) measurement was performed over Escalab250Xi (Thermo Scientific). The binding energies were calibrated internally by adventitious carbon deposit C(1s) with E_b = 284.8 eV. Thermogravimetric (TG) analysis was conducted on TGA-55 (Netzsch) under Ar from room temperature to 800 °C with heating rate of 10 °C min^{-1}.

Assembling and testing of single cells

Single cells were assembled in an Ar-filled glove box with oxygen and water concentrations lower than 0.1 ppm. A mixture of MgO and LiCl-LiBr-LiF eutectic salt with a mass ratio of 1:1 was used as the separator. The cathode materials were prepared by mechanically mixing transition metal disulfide, separator, and graphene in a mass ratio of 80:20:1. A total of 0.25 g cathode material and 0.45 g of separator were pressed into a 20 mm cathode/separator composite pellet under 20 MPa. A Li-B pellet (60 wt.% Li) with a thickness of 0.5 mm and a diameter of 19.5 mm was used as the anode. The performances of single cells were tested on a single-channel programmable electronic load (IT8511 plus). Unless otherwise stated, the cells were

tested at 500 °C with current densities of 200-500 mA cm^{-2} using a constant current model. The specific capacity was calculated based on the mass of transition metal disulfides in the cathode material.

Supporting figures

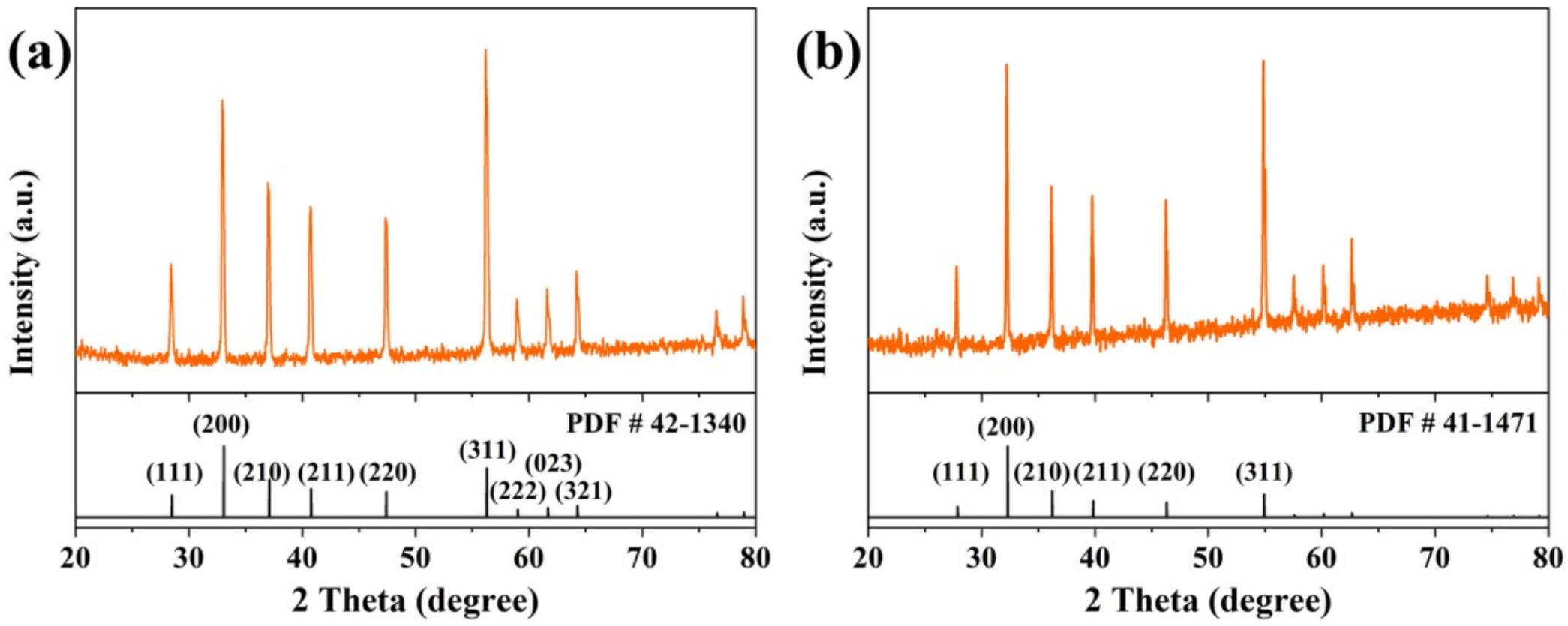

Fig. S1 XRD patterns of (a) FeS_2 and (b) CoS_2 prepared via the sulfidation of Fe and Co powder, respectively.

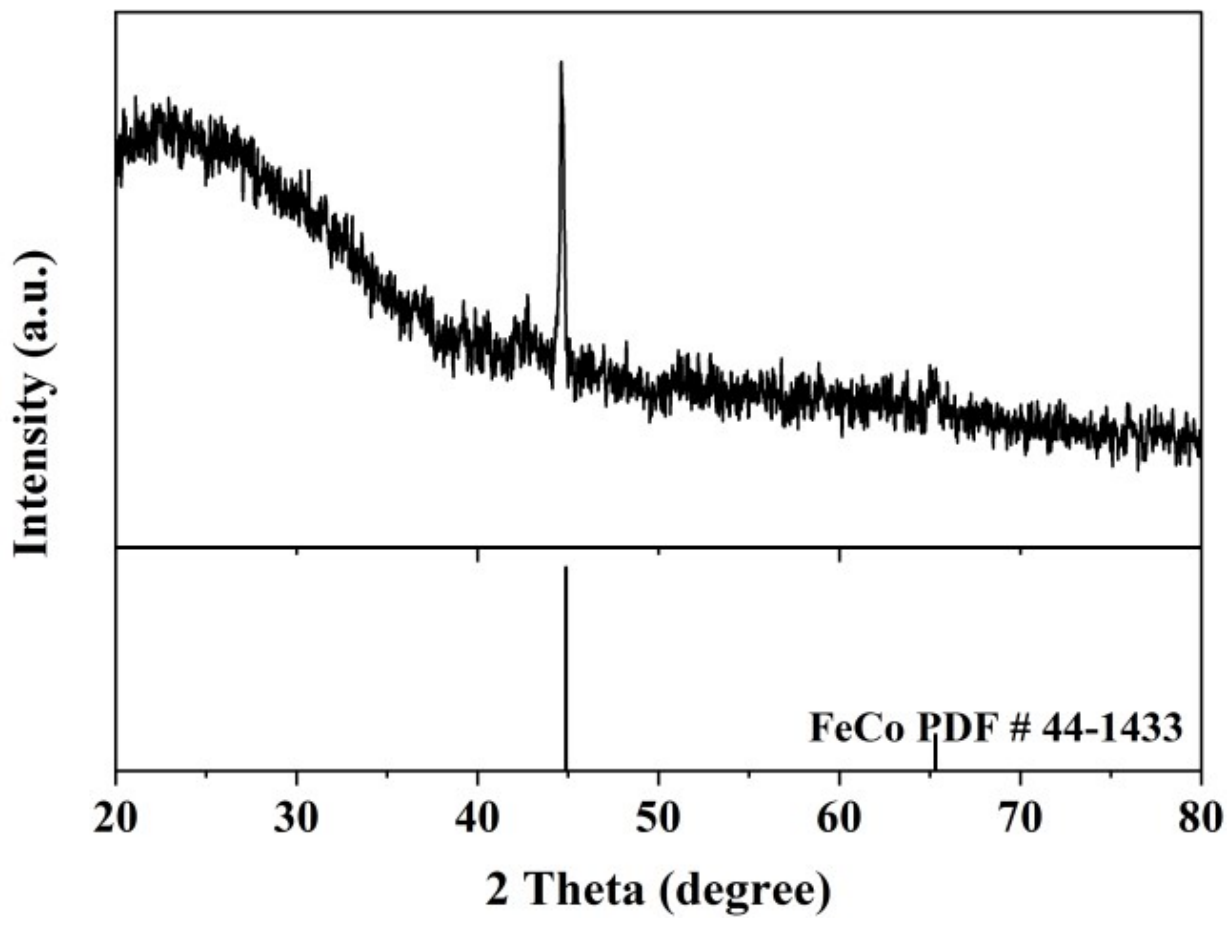

Fig. S2 XRD pattern the starting Fe-Co alloy.

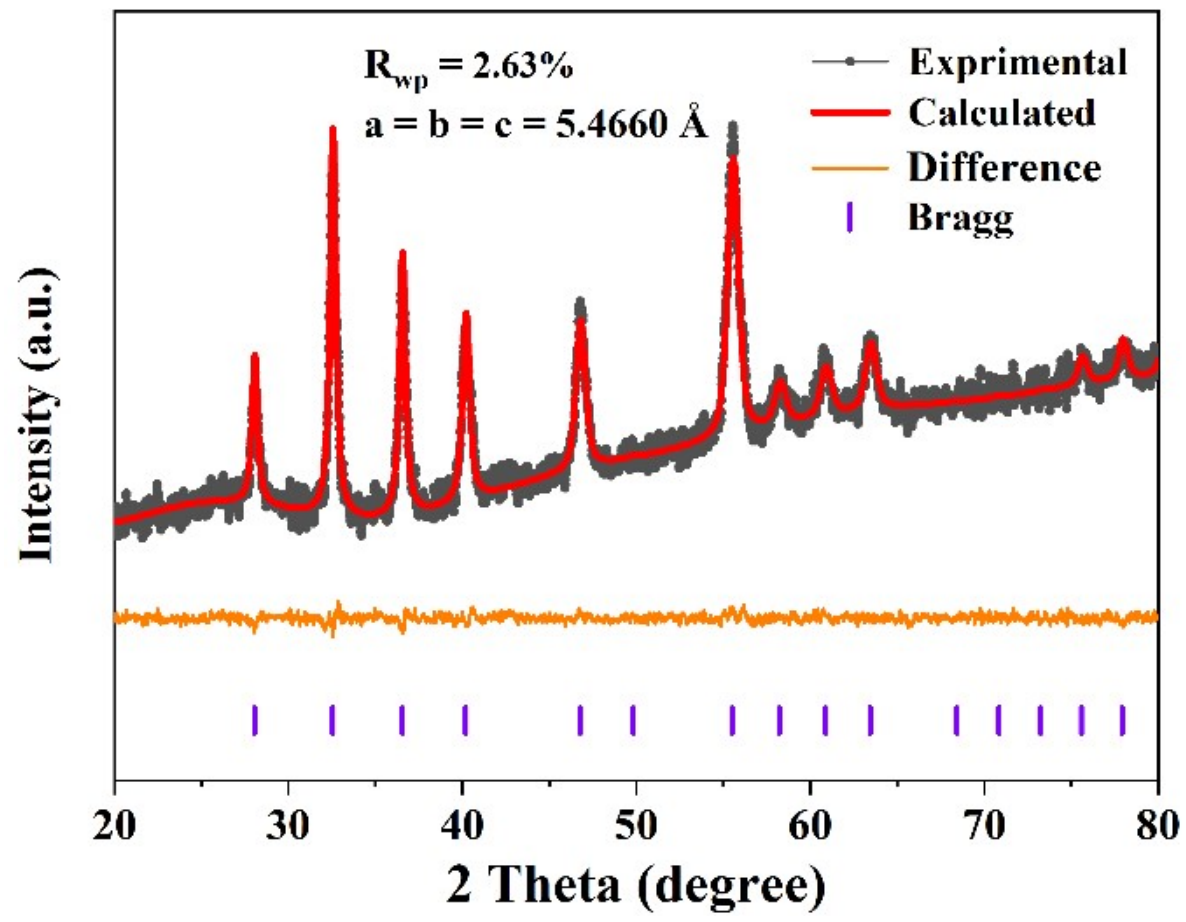

Fig. S3 XRD Rietveld refinement of $Fe_{0.5}Co_{0.5}S_2$.

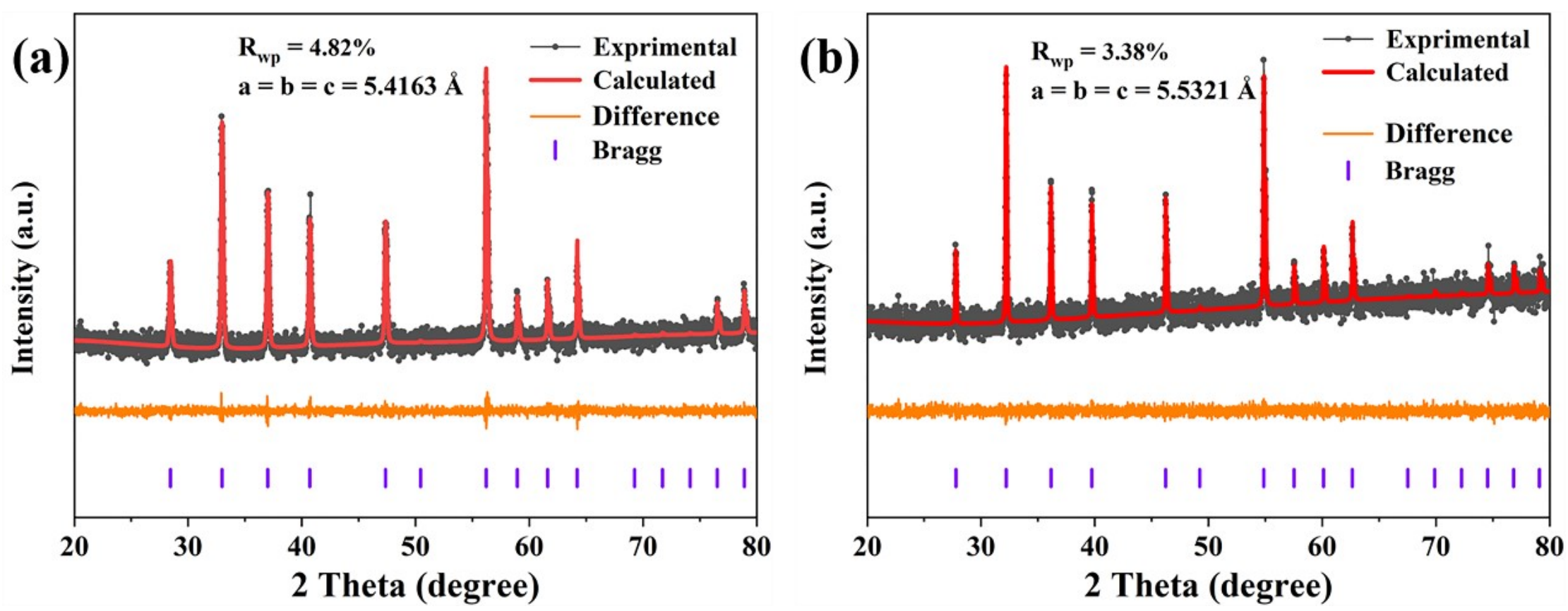

Fig. S4 XRD Rietveld refinement of (a) FeS_2 and (b) CoS_2.

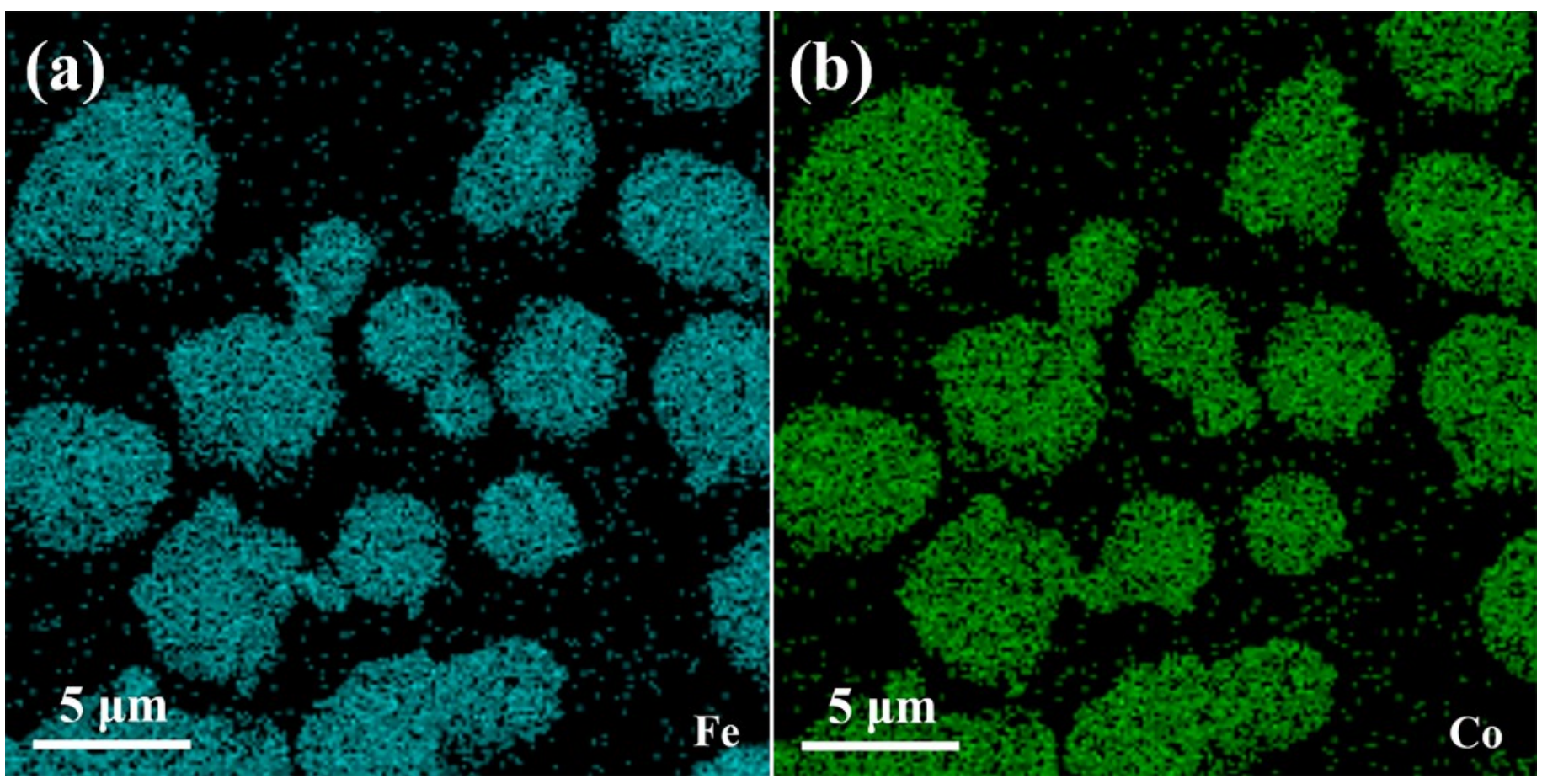

Fig. S5 SEM EDS-mapping images of Fe-Co alloy.

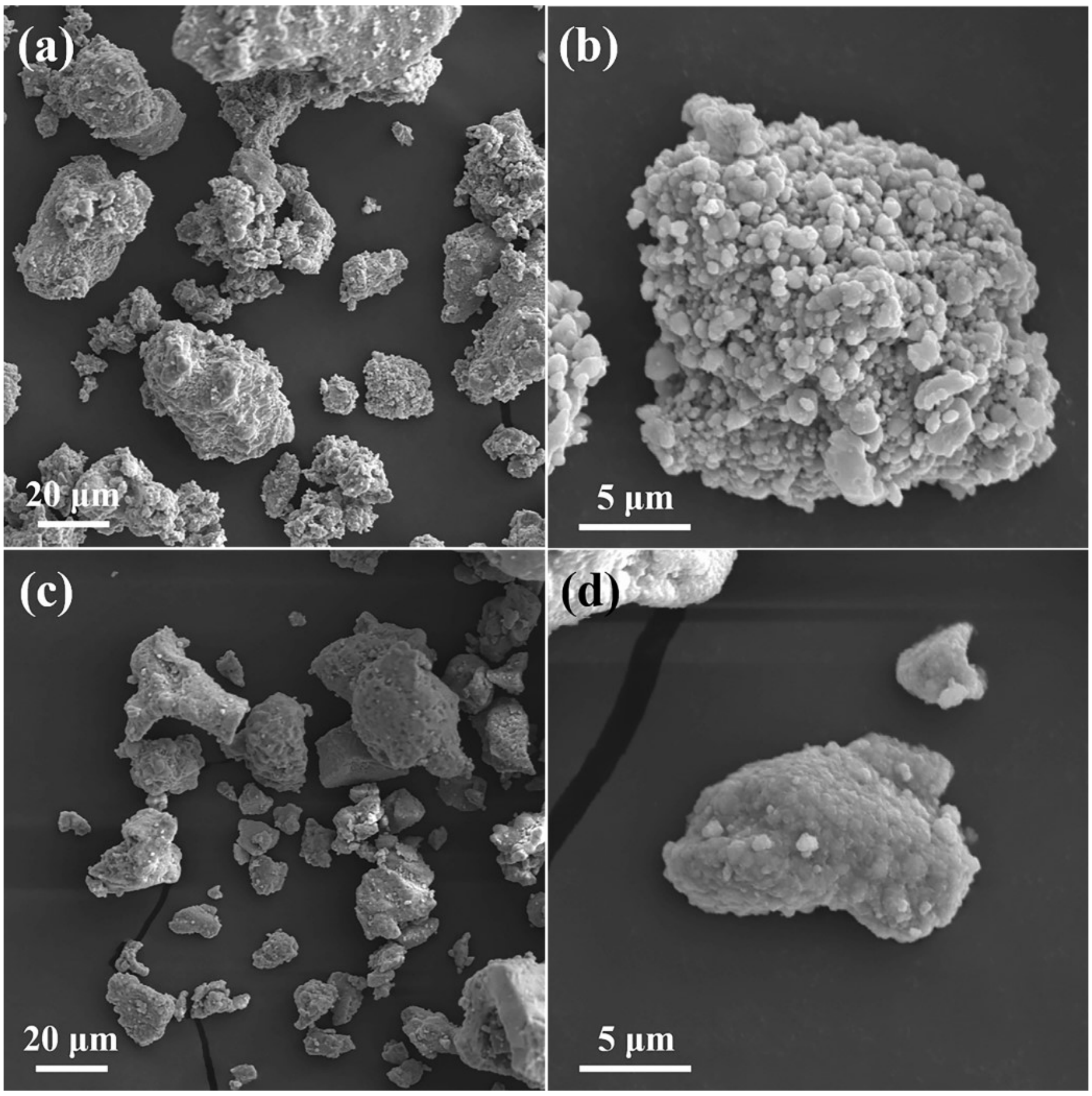

Fig. S6 SEM images of (a, b) FeS_2 and (c ,d) CoS_2 prepared via the sulfidation of Fe and Co powder, respectively.

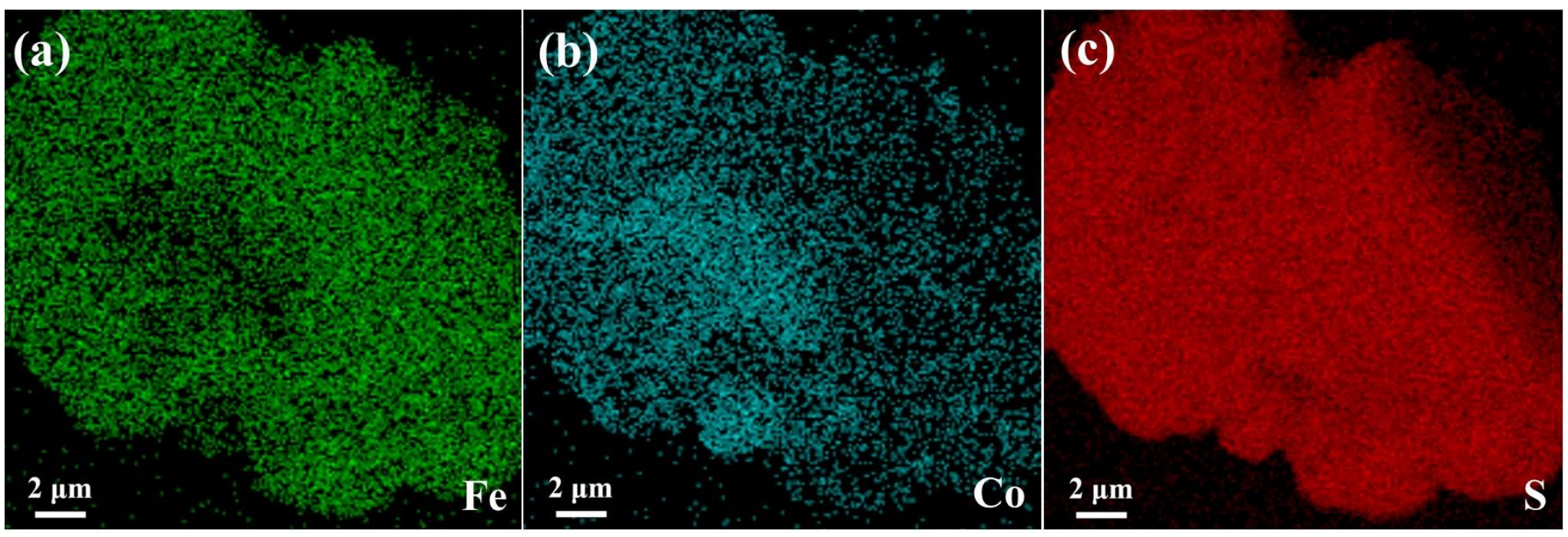

Fig. S7 SEM EDS-mapping images of $Fe_{0.5}Co_{0.5}S_2$.

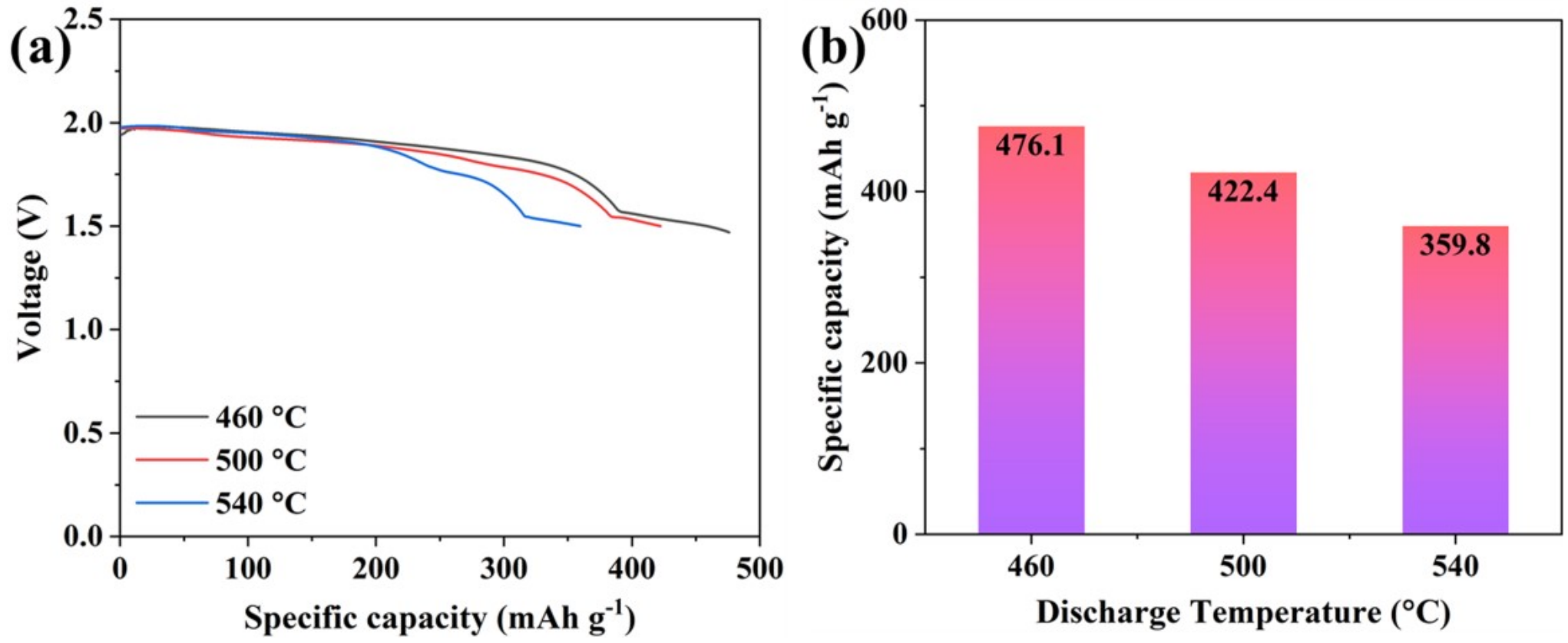

Fig. S8 (a) Discharge curves and (b) specific capacity of FeS_2/CoS_2 at different temperature (current density 100 mA cm^{-2}, cutoff voltage 1.5 V).

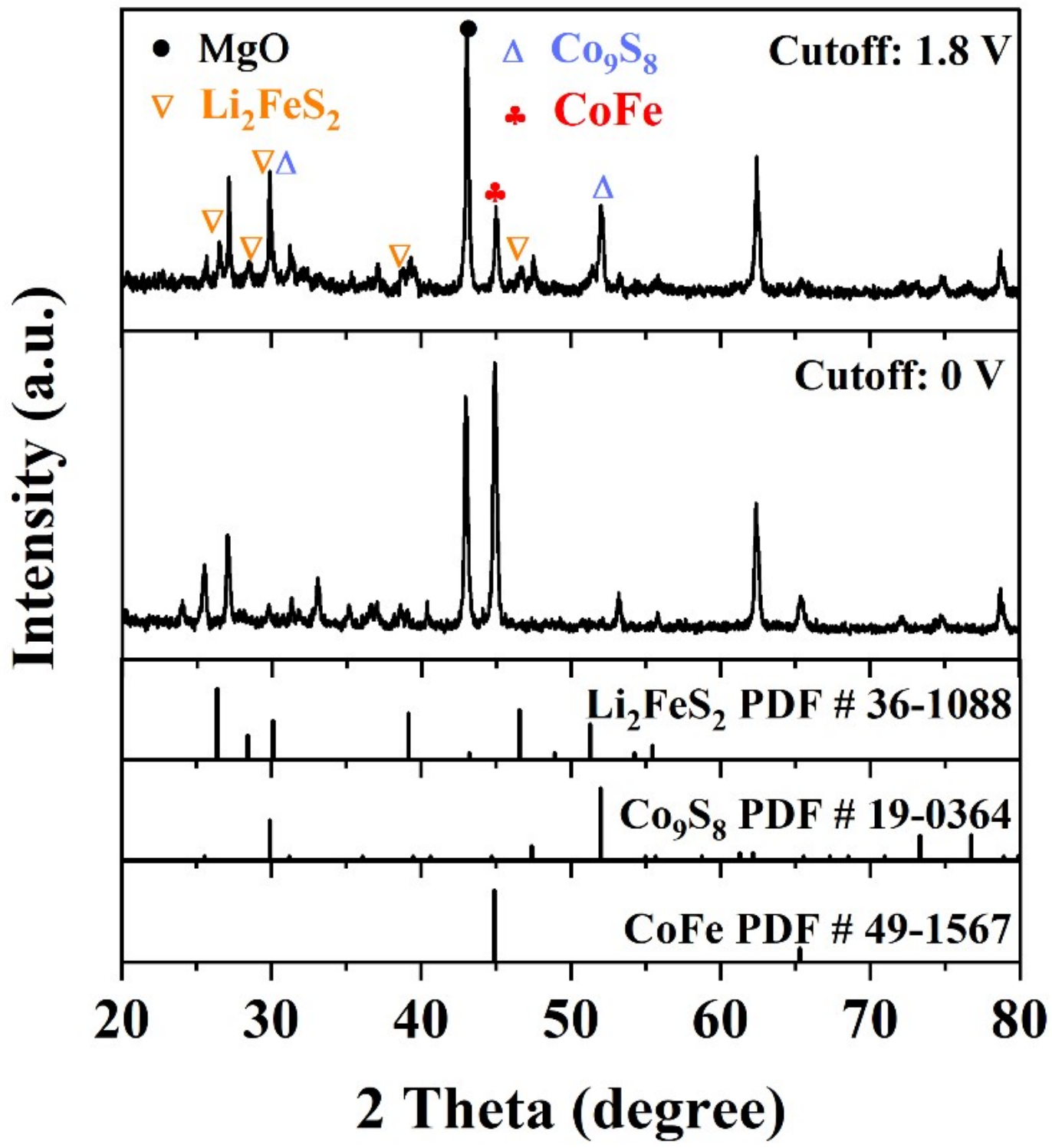

Fig. S9 XRD patterns of $Fe_{0.5}Co_{0.5}S_2$ after discharge.

Table S1 Comparison of thermal battery performances of selective reported transition metal disulfides tested at 500 °C with the cutoff voltage of 1.5 V.

Material	Synthesis method	Current density (mA cm^{-2})	Capacity (mAh g^{-1})	Ref.
$Fe_{0.5}Co_{0.5}S_2$	mechanical mixing and sintering	100	548	this work
Nano NiS_2	hydrothermal	200	484	[1]
$Ni_{0.95}Co_{0.05}S_2$	hydrothermal	100	526	[2]
$Ni_{0.5}Co_{0.5}S_2$	hydrothermal	100	434	[3]
NiS_2	ball-milling and sintering	100	502	[4]
L-$Fe_{0.5}Co_{0.5}S_2$	ball-milling and sintering	300	411	[5]
NiS_2@C	ball-milling and sintering	100	549	[6]
$Fe_{0.5}Co_{0.5}S_2$	ball-milling and sintering	100	385	[7]
FeS_2@CoS_2	hydrothermal and high-temperature vulcanization	500	251	[8]
NiS_2@g-C_3N_4	solid-state combustion	100	435	[9]
Nanostructured NiS_2	ball-milling and sintering	100	509	[10]

References

[1] C. Xu, C. Jin, X. Gong, X. Wang, S. Xie and Y. Fan, *J. Energy Storage*, **2022**, 45, 103742.

[2] X. Meng, H. Liu, S. Bi, C. Yang, S. Fan and L. Cao, *Electrochim. Acta*, **2023**, 464, 142879.

[3] H. Guo, L. Tang, Q. Tian, Y. Chu, B. Shi, X. Yin, H. Huo, X. Han, C. Yang, C. Wang, K. Tang, C. Wang, X. Zhang, J. Wang, L. Kong and Z. Lu, *ACS Appl. Mater. Interfaces*, **2020**, 12, 50377.

[4] W. Yang, L. Zhou, Z. Luo, J. Zhu, W. Yang, D. Li and L. Fu, *Adv. Eng. Mater.*, **2020**, 22, 2000299.

[5] J. Hu, L. Zhao, Y. Chu, Q. Tian, J. Wang, Y. Li, Q. Wu and Y. Zhu, *J. Alloy Compd.*, **2018**, 762, 109.

[6] C. Jin, L. Fu, J. Zhu, W. Yang, D. Li and L. Zhou, *J. Mater. Chem. A*, **2018**, 6, 7123.

[7] Q. Tian, Z. Yu, Y. Wu and H. Liu, *J. Mater. Sci.: Mater. Electron.*, 2022, **33**, 13968.

[8] Q. Tian, J. Hu, S. Zhang, X. Han, H. Guo, L. Tang, J. Wang and W. Hu, *Nanomaterials*, 2022, **12**, 1360.

[9] X. Meng, H. Liu, S. Bi, X. Geng, Q. Wang, S. Fan and L. Cao, *J. Phys. Chem. C*, 2022, **126**, 21550.

[10] C. Jin, L. Zhou, L. Fu, J. Zhu, D. Li and W. Yang, *J. Power Sources*, 2017, **352**, 83.
